# Persistent shortfalls in government health expenditure: a decade-long analysis of Abuja 15% target attainment across 52 African countries, 2014–2023

**DOI:** 10.1186/s41182-026-01102-6

**Published:** 2026-10-19

**Authors:** Muhammed Adeniyi Adebisi, Isaac Olushola Ogunkola, Don Eliseo Lucero-Prisno, Anas Ali Alhur, Quadri Aishat Adeola, Azeezat Fajembola, Goodness Ogeyi Odey, Nafisat Dasola Jimoh, Atunde Ahmed Olarewaju, Luke Alade, Joshua Aluga Gideon, Yusuff Adebayo Adebisi

**Affiliations:** 1 Faculty of Economics and Management Sciences, University of Ibadan, Ibadan, Nigeria; 2 Department of Economics, Lead City University, Ibadan, Nigeria; 3 Bristol Medical School, University of Bristol, Bristol, UK; 4 Department of Global Health and Development, London School of Hygiene and Tropical Medicine, London, UK; 5 Department of Health Informatics, College of Public Health and Health Informatics, University of Hail, Hail, Saudi Arabia; 6 Faculty of Pharmacy, Obafemi Awolowo University, Ile-Ife, Nigeria; 7 Women in Global Health, Abuja, Nigeria; 8 Department of Health Policy, London School of Economics and Political Science, London, UK; 9 London School of Hygiene and Tropical Medicine, London, UK; 10 African Young Leaders for Global Health, Abuja, Nigeria; 11 University of Ilorin, Ilorin, Nigeria; 12 Clinton Health Access Initiative, Abuja, Nigeria; 13 Equicare Strategies, Zaria, Nigeria; 14 College of Social Sciences, University of Glasgow, Glasgow, UK; 15 Faculty of Pharmacy, University of Ibadan, Ibadan, Nigeria; 16 Global Health Focus, Kigali, Rwanda

**Keywords:** Abuja Declaration, Health financing, Government health expenditure, Universal health coverage, Africa, Fiscal space

## Abstract

**Background:**

In 2001, African governments committed through the Abuja Declaration to allocating at least 15% of annual government budgets to health. More than 2 decades later, the extent to which this commitment has been achieved consistently, rather than in isolated years, remains unclear. We assessed recent trends, persistence of attainment, country trajectories and convergence in government prioritisation of health among the original Abuja Declaration endorsers.

**Methods:**

We conducted a longitudinal country-level (ecological) panel analysis of 52 original Abuja Declaration endorsers with complete comparable data from 2014 to 2023, supplemented by an analysis of all available data for 2001–2023. Annual data on domestic general government health expenditure as a percentage of general government expenditure were obtained from the World Bank World Development Indicators, drawing on the World Health Organisation Global Health Expenditure Database. Attainment was defined as an expenditure share of at least 15%. We summarised annual and subregional distributions, persistence of attainment and country-specific linear trends. Overall temporal change was examined using country fixed-effects regression with standard errors clustered by country. Exploratory beta- and sigma-convergence analyses, including specifications robust to regression to the mean, assessed whether lower-spending countries improved faster and whether between-country dispersion narrowed. Sensitivity analyses addressed classification thresholds and trend heterogeneity.

**Results:**

The unweighted mean government health-expenditure share increased from 6.73% in 2014 to 7.09% in 2023, while the median increased from 5.91 to 6.05%. Only South Africa met the 15% benchmark in 2023. Across the 10-year period, 48 countries (92.3%) never attained the benchmark, 3 (5.8%) attained it occasionally, and only South Africa demonstrated sustained attainment. Southern Africa had the highest mean share in 2023 at 13.79%, whereas Western Africa had the lowest at 5.87%. The average within-country linear trend was small and statistically uncertain at 0.053 percentage points per year (95%CI −0.052 to 0.157; *p* = 0.316). Country-specific trends varied substantially (standard deviation of slopes 0.32 percentage points per year; *p* < 0.001). Apparent catch-up by lower-baseline countries (*β* = −0.382; 95%CI −0.667 to −0.097) was not robust to regression to the mean, and dispersion did not consistently narrow. Over 2001–2023, 47 countries never attained the benchmark and the average within-country trend was 0.012 percentage points per year (95%CI −0.032 to 0.057).

**Conclusions:**

Government prioritisation of health among original Abuja Declaration endorsers remained substantially below the 15% benchmark during 2014–2023. More than nine in ten countries never attained the target, and only one demonstrated sustained attainment. This stagnation is long-standing, and since 2001, the continental distribution has barely moved. The Abuja benchmark should be accompanied by stronger accountability mechanisms and complementary measures of absolute spending adequacy, fiscal capacity and financial protection.

**Supplementary Information:**

The online version contains supplementary material available at https://doi.org/10.1186/s41182-026-01102-6.

## Background

In April 2001, at a Special Summit of the Organisation of African Unity in Abuja, African heads of state and government pledged to set a target of allocating at least 15% of their annual national budgets to the improvement of the health sector [1]. The Abuja Declaration became one of the most frequently invoked political commitments in global health. It emerged in the same year as the report of the Commission on Macroeconomics and Health, which argued that substantially increased public investment in health was both affordable and economically productive for low-income countries [2]. Together, these landmarks established domestic public financing as the cornerstone of sustainable health system development in Africa.

A decade after the pledge, the World Health Organisation reported that only a small minority of African countries had reached the 15% threshold, and that several had moved backwards [3]. The intervening years have strengthened the conceptual case for the commitment. Adequate and stable public financing is central to universal health coverage, because it enables pooling, reduces reliance on out-of-pocket payment, and allows purchasing that is aligned with population need [4]. Analyses of spending requirements for universal health coverage suggest that most African countries cannot finance an essential package of services without a marked increase in the priority given to health within government budgets [5]. Global health financing studies consistently show that government health spending in sub-Saharan Africa has grown mainly because economies and total budgets have grown, not because health has claimed a larger share of public resources [6, 7].

Despite the prominence of the Abuja benchmark, recent, systematic and comparable evidence on its attainment is limited. Existing assessments largely predate 2015, rely on snapshots of single years, or examine regional aggregates that obscure country trajectories [3, 8]. Two recent studies have revisited the question. Uzor et al. analysed government health expenditure across 53 African Union Member States for 2000–2022, combining trend analysis with a political-economy assessment of its determinants, and found that only South Africa met the benchmark in 2022 [9]. Asamani et al. developed a multicriteria index of vulnerability to reductions in official development assistance across 47 countries of the WHO African Region [10]. Neither study examined the persistence of attainment, formally estimated within-country trends or assessed convergence among the governments that made the 2001 commitment.

Three gaps therefore remain. First, attainment has not been evaluated across a recent, complete balanced panel restricted to the governments that actually made the 2001 commitment, with formal inference on within-country change. Second, the persistence of attainment has been neglected: a country that touches 15% once differs fundamentally, in policy terms, from one that sustains it. Third, little is known about whether country spending patterns are converging or diverging, a question that determines whether continental strategies should target systemic constraints or a handful of laggards. These questions have acquired new urgency, since the 2019 Africa Leadership Meeting Declaration renewed continental commitments to increased domestic health financing [11], the United Nations political declaration on universal health coverage reaffirmed the primacy of public funding [12], and the COVID-19 pandemic first expanded and then squeezed fiscal space for health.

This study addresses these gaps. Using internationally harmonised expenditure data from 2014 to 2023, it quantifies the extent to which the 52 original Abuja endorsers with comparable data prioritised health within government spending, evaluates progress towards and persistence of benchmark attainment, compares subregions, characterises each country’s policy trajectory, formally tests for temporal trends, heterogeneity in trends and convergence between countries, and places these findings within the full post-Declaration period from 2001 to 2023. This analysis is particularly timely as the Abuja Declaration reaches its twenty-fifth anniversary in 2026, providing an opportunity to assess whether sustained progress has followed a generation of political commitment.

## Methods

### Study design

We conducted a longitudinal, country-level panel analysis of government health expenditure across Africa from 2014 to 2023, with the country-year as the unit of analysis. In epidemiological terminology, this is an ecological design, because observations refer to national aggregates rather than to individuals [13]; the term describes the level of observation, not a particular estimator. The study assessed the extent to which African governments prioritised health within total government expenditure, progress towards the Abuja Declaration benchmark, the persistence of benchmark attainment, differences between African subregions, and whether spending patterns converged across countries over time.

### Data source

Annual data were obtained from the World Bank World Development Indicators for indicator SH.XPD.GHED.GE.ZS, defined as domestic general government health expenditure as a percentage of general government expenditure [14]. The analytical extract was downloaded on 25 July 2026. The underlying source is the World Health Organisation Global Health Expenditure Database, which provides internationally comparable annual estimates derived from national health accounts, government expenditure records and other official statistics, validated with Member States and structured according to the System of Health Accounts 2011 framework [15, 16]. The World Bank reports that the series was supplied by WHO and updated on 12 December 2025.

The 2014–2023 window was prespecified as the primary period for three reasons. First, it is the most recent decade for which all 52 original endorsers have complete data; earlier series are incomplete for some countries (the Somalia series begins in 2013 and the Zimbabwe series in 2010), so a longer primary window would have required an unbalanced panel or imputation. Second, it largely postdates the periods covered by previous continental assessments [3, 8] and spans the Sustainable Development Goal era, the 2019 Africa Leadership Meeting commitments [11] and the COVID-19 pandemic, and therefore describes the recent performance most relevant to current policy. Third, a fixed, recent window allows attainment and persistence to be judged on identical terms for every country. To place the findings in the context of the full period since the Declaration, we additionally analysed all available annual values for 2000–2023 from the same source.

### Measurement of government prioritisation of health

The principal measure was domestic general government health expenditure expressed as a percentage of general government expenditure for country i in year t. The numerator captures current health expenditure financed from domestic public sources and the denominator captures total general government expenditure, so the measure reflects the share of actual government spending devoted to health. This indicator is not identical to the proportion initially appropriated to health in an approved national budget, to which the Abuja Declaration formally refers. It was therefore interpreted as the closest internationally comparable operational measure of government prioritisation of health relative to the Abuja benchmark, rather than as a direct measure of enacted budget allocations. For each country-year, attainment was defined as an expenditure share of at least 15%; values below 15% were classified as not attaining the benchmark.

### Study population

The primary analytical population comprised the original endorsers of the 2001 Abuja Declaration. The source roster contained 53 original endorsers; the Sahrawi Arab Democratic Republic had no separate standardised expenditure series in the World Bank and WHO data and was excluded from numerical analyses. The final primary sample comprised 52 countries, each with complete observations for all ten years, yielding a strongly balanced panel of 520 country-year observations with no missing values and no imputation. Morocco and South Sudan were not original 2001 endorsers and were excluded from the primary analysis. Descriptive sensitivity analyses using current African Union membership, where data were available, produced consistent conclusions; the original endorsers constituted the prespecified primary population, because the study evaluated progress among the governments to which the original commitment applied.

### Subregions and descriptive analysis

Countries were grouped into Eastern, Middle, Northern, Southern and Western Africa using the United Nations Statistics Division M49 classification [17] (country membership in Supplementary Table S7). Annual distributions were summarised with the unweighted country-level mean, median, standard deviation and interquartile range, and with the number and percentage of countries meeting the 15% benchmark each year. Subregional means and medians were calculated for 2014 and 2023, together with the mean within-country change in percentage points. All descriptive estimates gave each country equal weight, irrespective of population or economic size, because the research question concerned the proportion of governments meeting a political commitment rather than the expenditure experience of the average African resident.

### Persistence and 2023 performance classification

For each country, we counted the years during 2014–2023 in which the expenditure share was at least 15% and the longest run of consecutive attainment years. Persistence was classified as never attained (no years), occasional attainment (1–3 years), repeated attainment (4–7 years) or sustained attainment (at least 8 years); the repeated-attainment category proved empty and was omitted from the results tables. A policy-oriented 2023 classification combined the latest expenditure share with the country-specific linear trend over 2014–2023, estimated from separate ordinary least-squares regressions of the share on calendar year. Countries were classified as met Abuja target (2023 share of at least 15%); near target (12 to below 15%); below target and improving (below 12% with a trend of at least +0.20 percentage points per year); below target and broadly stagnant (below 12% with a trend between −0.20 and +0.20); or below target and declining (below 12% with a trend of −0.20 or lower). The 12% and ±0.20 thresholds were investigator-defined descriptive cut points intended to distinguish materially different trajectories, not inferential thresholds.

The 12% cut point corresponds to 80% of the benchmark, within 3 percentage points of the target, and ±0.20 percentage points per year corresponds to a cumulative change of about 2 percentage points over the decade, roughly one-quarter of the average 2023 distance to the target (7.9 points) and about half the between-country standard deviation of country-specific slopes. Because these cut points were nonetheless investigator-defined, we assessed the sensitivity of the classification to alternative near-target thresholds (10 and 13%), alternative trend thresholds (± 0.10 to ±0.30 percentage points per year), alternative trend estimators (the Theil–Sen slope and the difference between 2021–2023 and 2014–2016 averages), an inferential rule based on whether the 95% confidence interval of each country slope excluded zero, and the use of 2022 rather than 2023 as the reference year. Agreement with the primary classification was summarised by percentage agreement and Cohen’s kappa. We also compared the classification with quintiles of the 2023 share and of country-specific trends, which are distribution-based but not anchored to the benchmark.

### Longitudinal trend analysis

The overall association between calendar year and the expenditure share was examined with country fixed-effects linear regression, in which the year coefficient represents the average within-country annual percentage-point change and standard errors were clustered by country to account for serial correlation and heteroskedasticity. A second fixed-effects model treated calendar year as categorical with 2014 as reference; an omnibus Wald test assessed whether the year indicators were jointly zero, and model-based annual marginal means with 95% confidence intervals were estimated for graphical presentation. To examine whether prioritisation differed during and after the onset of the COVID-19 pandemic, a fixed-effects model restricted from 2017 to 2023 compared 2020 to 2023 with 2017 to 2019. This comparison was interpreted descriptively rather than causally, because the design lacked a counterfactual comparison group.

Because the fixed-effects year coefficient summarises the average within-country trend and may conceal heterogeneous trajectories, we assessed heterogeneity in two ways: an F test of equality of country-specific slopes in a model with country-by-year interactions, and a linear mixed model with country-level random intercepts and random slopes for calendar year, estimated by maximum likelihood and compared with a random-intercept model using a likelihood-ratio test referred to a 50:50 mixture of Chi-square distributions. We further estimated trends by subregion, by fragile and conflict-affected status according to the World Bank list for fiscal year 2024 [18], and by natural-resource intensity according to the International Monetary Fund classification [19], using interactions between calendar year and group in fixed-effects models with country-clustered standard errors (country membership in Supplementary Table S7). Finally, because some annual values were identical to the preceding year’s value, consistent with carry-forward estimation where recent country reports were unavailable, we re-estimated the linear trend after excluding such observations.

### Convergence analysis

Two complementary approaches assessed whether between-country differences narrowed over time [20]. Beta convergence regressed each country’s absolute change from 2014 to 2023 on its 2014 share using ordinary least-squares regression with heteroskedasticity-robust standard errors; a negative coefficient indicates that countries starting lower experienced larger increases. This analysis was treated as exploratory, because change-score models are susceptible to mathematical coupling and regression to the mean. Sigma convergence regressed the annual cross-country standard deviation and interquartile range on calendar year with robust standard errors; a negative coefficient indicates narrowing dispersion. These models rested on ten annual observations and were likewise exploratory.

To address mathematical coupling and regression to the mean directly, we used three further approaches [21–23]: Oldham’s method, which correlates the change with the average of the initial and final values [21]; a regression of the 2014–2023 change on the mean 2011–2013 share, a baseline measured independently of the change score; and an instrumental-variable estimate in which the 2014 share was instrumented by the 2011–2013 mean. Because the sigma-convergence regressions rested on only ten annual observations, we also estimated country-resampling bootstrap confidence intervals (5,000 replicates) for the change in the cross-country standard deviation and interquartile range between 2014 and 2023, and extended the sigma-convergence regressions to 23 annual observations (2001–2023) with Newey–West standard errors.

### Longer-term analysis, 2001–2023

To assess whether the recent pattern reflected longer-run dynamics, we analysed all available annual values from 2000 to 2023. The year 2000 was used only as a pre-Declaration reference in figures. For 2001–2023, we summarised the annual distribution and the number of countries meeting the benchmark, counted years of attainment and the longest consecutive run of attainment for each country, and estimated the average within-country linear trend with country fixed effects and country-clustered standard errors in the 50 countries with complete data for all 23 years (Somalia and Zimbabwe were excluded from this model). A piecewise specification allowed the slope to differ before and after 2014.

### Statistical inference and software

All tests were two-sided with significance evaluated at the 5% level, and estimates are presented with 95% confidence intervals. No adjustment was made for multiple testing, because the models addressed distinct but complementary aspects of temporal change, persistence and convergence; p values were interpreted alongside effect sizes and the exploratory status of secondary analyses. Analyses used StataNow version 19.5 [24] and Python 3.11 with the statsmodels and linearmodels libraries. Analyses were based on unrounded values, with tabulated values generally presented to two decimal places.

### Ethical considerations

The study used publicly available, aggregate country-level data with no individual-level or identifiable information; research ethics approval and informed consent were not required.

## Results

### Overall level and trend of government prioritisation of health

**Fig. 1:**
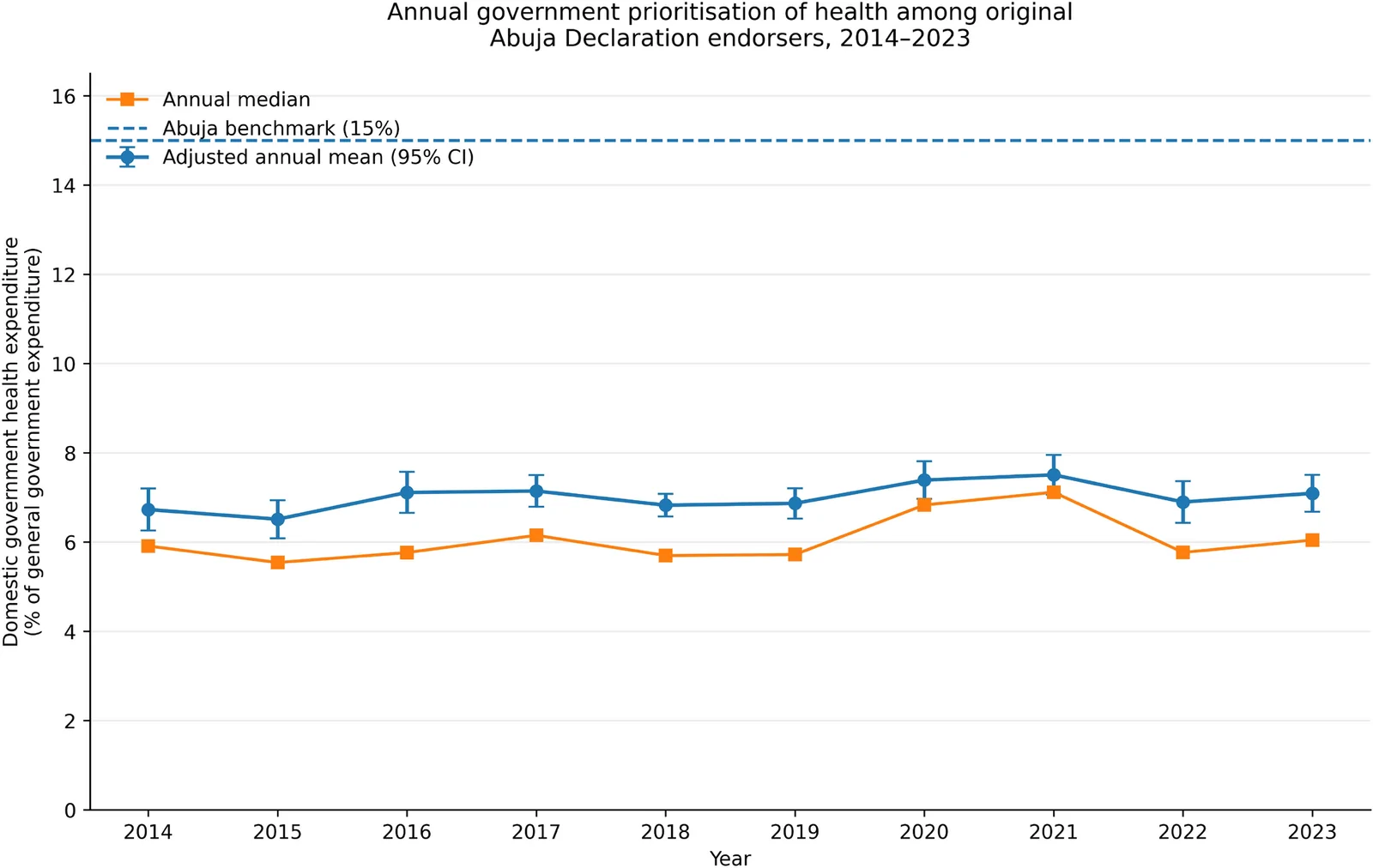
Annual government prioritisation of health among original Abuja Declaration endorsers, 2014–2023. Points and error bars show the adjusted annual mean domestic general government health expenditure as a percentage of general government expenditure, with 95% confidence intervals estimated from a country fixed-effects model with standard errors clustered by country. The second line shows the annual country-level median. The horizontal dashed line represents the Abuja benchmark of 15%

Across the 520 country-year observations, government prioritisation of health remained far below the Abuja benchmark throughout the study period. The unweighted mean expenditure share was 6.73% in 2014 and 7.09% in 2023, and the median moved from 5.91 to 6.05% (Table 1). Model-adjusted annual means fluctuated within a narrow band of roughly 6.5–7.5%, peaking in 2021 before easing after 2022, and at no point approached the benchmark (Fig. 1). In the country fixed-effects model, the average within-country change was +0.053 percentage points per year (95%CI −0.052 to +0.157; *p* = 0.316), a pace that, even if maintained, would imply more than a century to close the average gap to 15% (Table 3). The categorical-year model detected modest overall variation across years (F (9, 51) = 2.10; *p* = 0.046), reflecting a temporary rise in 2020 and 2021 rather than sustained gains. The descriptive post-2020 comparison showed a mean share 0.275 percentage points higher in 2020 to 2023 than in 2017 to 2019, but this difference was compatible with chance (95%CI −0.210 to +0.761; *p* = 0.260).

**Table 1:** Government prioritisation of health among original Abuja Declaration endorsers, by African subregion, 2014–2023

		2014		2023		Change, 2014–2023	Attainment, 2023
African subregion	Countries, *n*	Mean (%)	Median (%)	Mean (%)	Median (%)	Mean change (pp)	Countries meeting 15%, *n* (%)
Eastern Africa	17	7.39	7.78	6.17	5.51	−1.22	0 (0.0)
Middle Africa	9	4.25	3.95	6.58	5.90	+2.33	0 (0.0)
Northern Africa	5	9.09	10.62	8.38	7.15	−0.71	0 (0.0)
Southern Africa	5	11.59	11.59	13.79	14.61	+2.20	1 (20.0)
Western Africa	16	5.17	4.89	5.87	5.27	+0.69	0 (0.0)
All original endorsers	52	6.73	5.91	7.09	6.05	+0.36	1 (1.9)

Values are unweighted country-level summaries of domestic general government health expenditure as a percentage of general government expenditure. Mean change is the average within-country percentage-point change from 2014 to 2023. Analyses include 52 original Abuja Declaration endorsers with comparable data; the Sahrawi Arab Democratic Republic had no separately reported standardised expenditure series

Data source: World Bank World Development Indicators, indicator SH.XPD.GHED.GE.ZS; underlying source, WHO Global Health Expenditure Database. pp, percentage points

The average trend concealed substantial variation between countries. In 2023, expenditure shares ranged from 2.37% in Eritrea to 16.89% in South Africa; the between-country standard deviation was 3.0 percentage points, and 78% of the total variance in the panel lay between rather than within countries. Country-specific slopes ranged from −1.47 (Madagascar) to +0.68 (Cabo Verde) percentage points per year (median +0.14; interquartile range −0.10 to +0.27). Both the test of equal country slopes (F (51, 416) = 8.33; *p* < 0.001) and the random-slope mixed model (standard deviation of country slopes 0.32 percentage points per year; likelihood-ratio *p* < 0.001) confirmed that trends differed materially between countries. The mixed-model mean slope (+ 0.053; 95%CI −0.042 to +0.147) was identical to the fixed-effects estimate, so the latter should be read as the average of heterogeneous country trajectories rather than as a common trend. Trends differed by subregion (joint *p* < 0.001), being positive in Middle Africa (+ 0.34 percentage points per year; 95%CI +0.20 to +0.48) and Southern Africa (+ 0.29; +0.06 to +0.52) and negative but imprecise in Eastern (− 0.16; −0.39 to +0.08) and Northern Africa (− 0.17; −0.61 to +0.27). Trends did not differ significantly by fragility status (+ 0.10 in fragile and conflict-affected countries versus +0.02 in others; p for difference = 0.457) or by natural-resource intensity (+ 0.14 versus −0.03; *p* = 0.113). Excluding values identical to the preceding year did not materially alter the average trend (+ 0.070; 95%CI −0.039 to +0.179) (Supplementary Table S5).

### Benchmark attainment and persistence

**Fig. 2:**
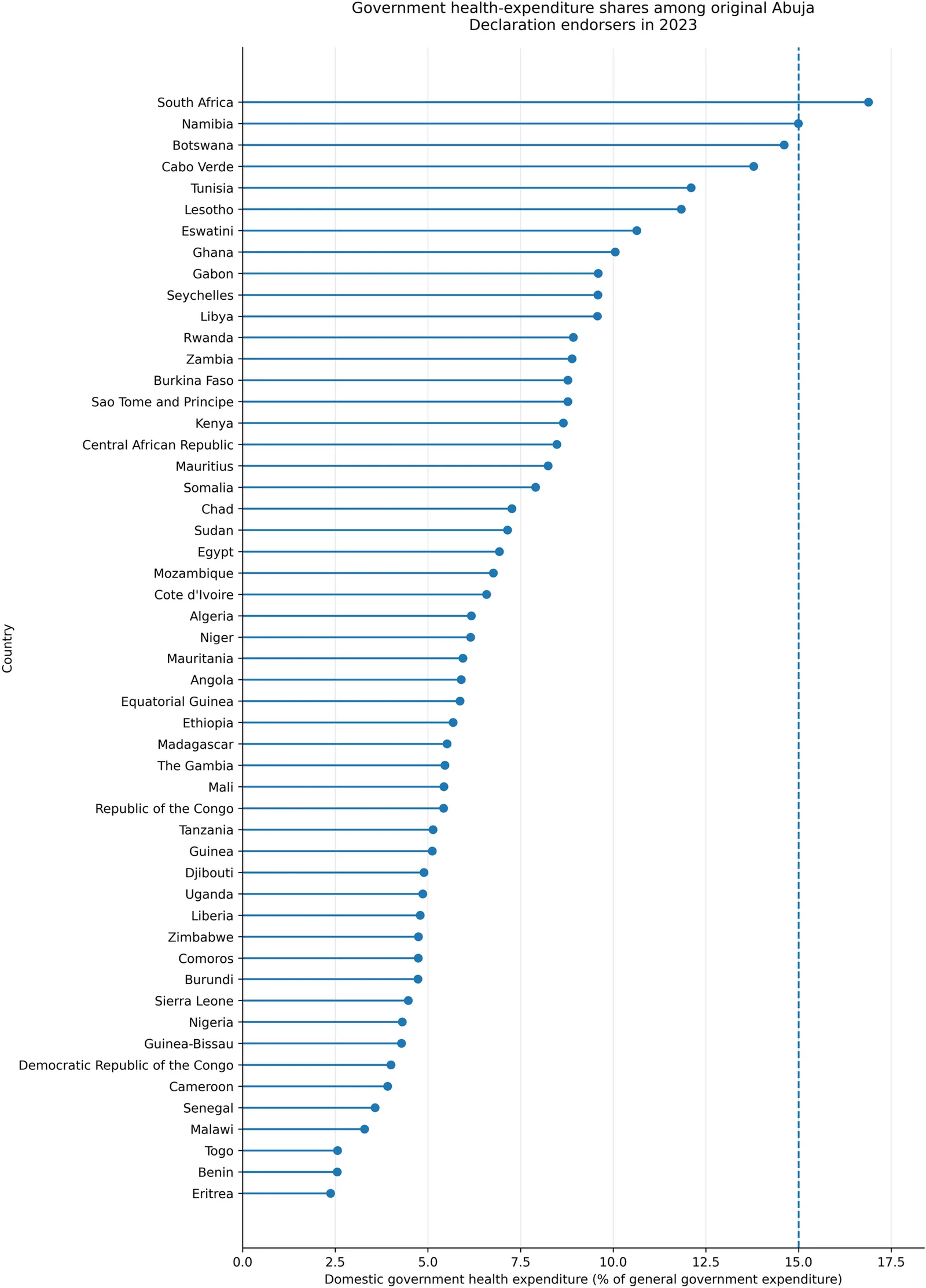
Government health-expenditure shares among original Abuja Declaration endorsers in 2023. Countries are ranked from highest to lowest domestic general government health expenditure as a percentage of general government expenditure. The vertical dashed line indicates the Abuja benchmark of 15%

**Fig. 3:**
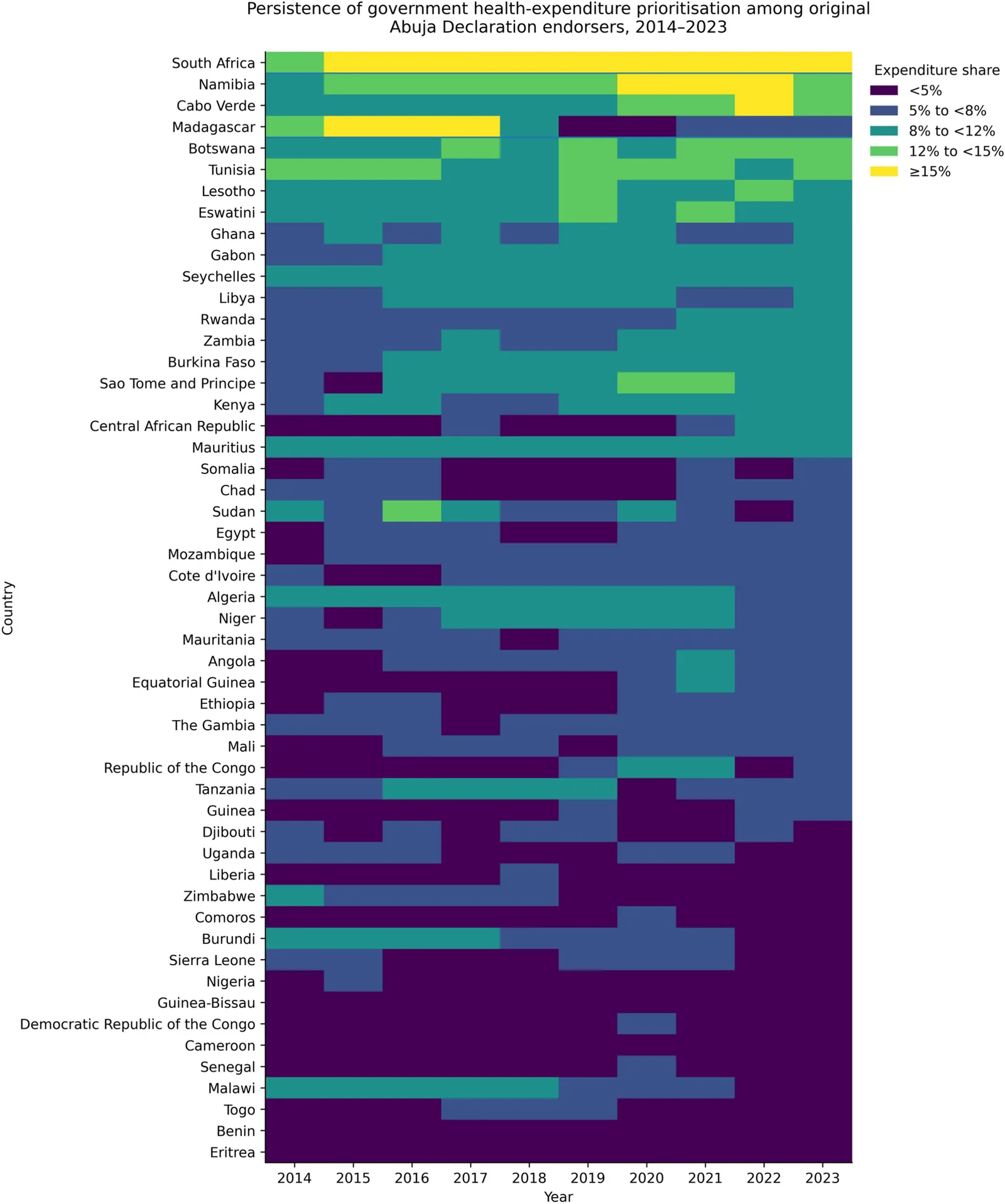
Persistence of government health-expenditure prioritisation among original Abuja Declaration endorsers, 2014–2023. Rows represent countries and columns represent years. Cell shading indicates domestic general government health expenditure as a percentage of general government expenditure, categorised as < 5%, 5% to < 8%, 8% to < 12%, 12% to < 15%, and ≥ 15%. Countries are ordered by persistence of Abuja target attainment (sustained attainment, occasional attainment and never attained) and then by 2023 expenditure share within each group

Attainment of the 15% benchmark was rare and fragile. No country met the benchmark in 2014, no more than three countries did so in any single year, with the maximum of three (5.8%) recorded in 2022, and only one country, South Africa, met it in 2023 with a share of 16.89% (Table 2 and Fig. 2, Supplementary Table S2). Over the full decade, 48 of 52 countries (92.3%) never reached the benchmark in any year. Three countries (5.8%), Namibia, Madagascar and Cabo Verde, attained it occasionally, in 1–3 years, and only South Africa (1.9%) sustained attainment, meeting the benchmark in nine consecutive years from 2015 (Table 2 and Fig. 3). No country fell in the repeated-attainment category of 4–7 years, indicating a sharp separation between one broadly sustained achiever and an overwhelming majority that never crossed the threshold. Madagascar illustrates the fragility of episodic attainment: after exceeding 15% from 2015 to 2017, its share collapsed to 5.51% by 2023, a fall of 8.41 percentage points, the largest decline in the sample (Supplementary Table S1).

**Table 2:** Persistence of Abuja target attainment and 2023 performance among original Abuja Declaration endorsers, 2014–2023

Assessment	Category	Definition	Countries, *n*	Percentage
Target attainment during 2014–2023	Never attained	Did not reach 15% in any year	48	92.3
	Occasional attainment	Reached 15% in 1–3 years	3	5.8
	Sustained attainment	Reached 15% in at least 8 years	1	1.9
Performance in 2023	Met Abuja target	2023 share ≥ 15%	1	1.9
	Near target	2023 share 12% to < 15%	4	7.7
	Below target, improving	2023 share < 12% and trend ≥ +0.20 pp/year	16	30.8
	Below target, broadly stagnant	2023 share < 12% and trend > −0.20 to < +0.20 pp/year	22	42.3
	Below target, declining	2023 share < 12% and trend ≤ −0.20 pp/year	9	17.3

Percentages are calculated within each assessment and are based on 52 original Abuja Declaration endorsers with comparable data. South Africa was the only sustained achiever; Namibia, Madagascar and Cabo Verde attained the target occasionally. Persistence was based on the number of years in which the expenditure share reached at least 15%. The 2023 performance classification combined the latest expenditure share with each country-specific linear trend during 2014–2023. Thresholds of ±0.20 percentage points per year were investigator-defined. pp, percentage points

### Subregional patterns

Subregional trajectories diverged markedly (Table 1). Southern Africa recorded the highest prioritisation, with the mean rising from 11.59% in 2014 to 13.79% in 2023 (mean within-country change +2.20 percentage points) and one of five countries meeting the benchmark in 2023. Middle Africa registered the largest mean improvement (+ 2.33 percentage points) but from the lowest base, rising from 4.25% to 6.58%. Western Africa improved modestly (+ 0.69 points, to a 2023 mean of 5.87%). By contrast, mean shares fell in Eastern Africa (− 1.22 points, from 7.39% to 6.17%) and Northern Africa (− 0.71 points, from 9.09% to 8.38%). Outside Southern Africa, no country in any subregion met the benchmark in 2023.

### Country performance in 2023

The 2023 policy classification underlines how distant most governments remain from the commitment (Table 2 and Fig. 2). Beyond South Africa, four countries (7.7%) were near the target with 2023 shares between 12 and 15%: Namibia (14.99%), Botswana (14.61%), Cabo Verde (13.79%) and Tunisia (12.10%). Sixteen countries (30.8%) were below 12% but improving at 0.20 percentage points per year or faster, including several starting from very low levels such as the Central African Republic (+ 4.53 points over the decade), Equatorial Guinea (+ 3.80) and the Republic of the Congo (+ 3.47). The largest single group, 22 countries (42.3%), was below target and broadly stagnant. Nine countries (17.3%) were below target and declining, among them Madagascar (− 8.41 percentage points), Zimbabwe (− 6.19), Malawi (− 5.29), Algeria (− 5.25) and Burundi (− 5.06). Country-level values, trends and classifications for all 52 countries are provided in Supplementary Tables S1 and S2, and the joint distribution of 2014 and 2023 values is shown in Supplementary Figure S1.

The classification was robust to reasonable alternative specifications (Supplementary Table S4). Agreement with the primary classification was 94% and 98% for near-target thresholds of 10% and 13% (*κ* = 0.92 and 0.97), 77% to 92% for trend thresholds between ±0.10 and ±0.30 percentage points per year (*κ* = 0.65 to 0.89), 94% and 96% for the Theil–Sen and endpoint-average trend estimators (*κ* = 0.92 and 0.94), 87% for the inferential rule (*κ* = 0.80) and 87% when 2022 was used as the reference year (*κ* = 0.81); under every specification, at least 23 countries (44%) were below target and not improving. Using 2022 as the reference year, three countries (South Africa, Namibia and Cabo Verde) met the benchmark, underlining how fragile attainment near the threshold is from one year to the next. All nine declining countries fell in the lowest quintile of country-specific trends and all 16 improving countries in the top two quintiles, whereas the top quintile of 2023 shares grouped countries at 9.6% with South Africa at 16.9%; a purely relative classification would therefore obscure distance from the commitment.

### Convergence between countries

Evidence of genuine convergence was weak once the mechanical dependence of change scores on baseline values was addressed (Table 3). In the conventional beta-convergence model, each additional percentage point of 2014 expenditure share was associated with a 0.382 percentage-point smaller change over the decade (95%CI −0.667 to −0.097; *p* = 0.010). However, this association attenuated by about one third and was no longer statistically significant when the change was regressed on an independently measured 2011–2013 baseline (*β* = −0.247; 95%CI −0.570 to +0.076; *p* = 0.131) or when the 2014 share was instrumented by that baseline (*β* = −0.253; 95%CI −0.554 to +0.047; *p* = 0.098), and Oldham’s method showed no association between change and average level (*r* = −0.02; 95%CI −0.29 to +0.25; *p* = 0.887). Part of the apparent catch-up therefore reflects regression to the mean. Dispersion measures were similarly inconclusive: the cross-country interquartile range narrowed by 0.114 percentage points per year (95%CI −0.214 to −0.015; *p* = 0.030), whereas the standard deviation was essentially unchanged (+ 0.001 per year; p = 0.933), bootstrap confidence intervals for the 2014–2023 change in both measures included zero (standard deviation −0.05, 95%CI −0.73 to +0.52; interquartile range −0.93, 95%CI −2.94 to +0.70), and neither measure trended over 2001–2023 (Supplementary Table S6). The joint pattern in Supplementary Figure S1, with most countries clustered near the 45-degree equality line and below both 15% reference lines, is therefore best described as a stable distribution centred on roughly 5–9%, less than half the Abuja commitment, rather than as convergence towards any common level.

**Table 3:** Longitudinal change and convergence in government prioritisation of health among original Abuja Declaration endorsers, 2014–2023, with robustness analyses

Analysis	Estimate or test statistic	95%CI	p value
Linear annual trend, percentage points per year	+0.053	−0.052 to +0.157	0.316
Linear annual trend, 2001–2023 (50 countries), percentage points per year	+0.012	−0.032 to +0.057	0.572
Heterogeneity of country-specific trends: SD of random slopes, percentage points per year	0.323	–	<0.001
Overall variation across calendar years	F(9, 51) = 2.10	–	0.046
Post-2020 period versus 2017–2019, percentage-point difference	+0.275	−0.210 to +0.761	0.260
Beta convergence: association between 2014 share and 2014–2023 change	−0.382	−0.667 to −0.097	0.010
Beta convergence with independent baseline: association between 2011–2013 mean share and 2014–2023 change	−0.247	−0.570 to +0.076	0.131
Oldham’s method: correlation between 2014–2023 change and mean of 2014 and 2023 shares	−0.020	−0.291 to +0.254	0.887
Sigma convergence: annual change in between-country SD	+0.001	−0.024 to +0.025	0.933
Sigma convergence: annual change in between-country IQR	−0.114	−0.214 to −0.015	0.030
Sigma convergence, 2001–2023 (50 countries): annual change in between-country SD	+0.001	−0.009 to +0.011	0.828
Sigma convergence, 2001–2023 (50 countries): annual change in between-country IQR	+0.030	−0.019 to +0.079	0.214

Analyses include 52 original Abuja Declaration endorsers with comparable data. Linear, categorical-year and post-2020 models used country fixed effects with standard errors clustered by country. The post-2020 estimate compares 2020–2023 with 2017–2019, and is descriptive rather than causal. Beta convergence was exploratory, because change-score models can be affected by mathematical coupling and regression to the mean. The independent-baseline and Oldham analyses address this. The heterogeneity p value is from a likelihood-ratio test comparing random-slope and random-intercept mixed models. Analyses for 2001–2023 exclude Somalia and Zimbabwe, whose series are incomplete, and use Newey–West standard errors for sigma convergence. Sigma convergence assessed changes in between-country dispersion

*CI* confidence interval, *IQR* interquartile range, *SD* standard deviation

### Longer-term context, 2001–2023

Extending the analysis to the full post-Declaration period reinforced these findings (Supplementary Table S3; Supplementary Figure S2). Among the 50 countries with complete data, the unweighted mean share was 6.88% in 2001 and 7.12% in 2023, while the median fell from 6.65% to 6.05%. The average within-country trend over 2001–2023 was +0.012 percentage points per year (95%CI −0.032 to +0.057; *p* = 0.572); in the piecewise model the slope was −0.037 (95%CI −0.103 to +0.029) before 2014 and +0.096 (+ 0.002 to +0.191) from 2014 onwards, a modest change that remains far too small to approach the benchmark. No more than three countries met the benchmark in any year between 2001 and 2023, and 47 of 52 countries (90.4%) never met it in any year with available data. Namibia illustrates reversal: it exceeded 15% in every year from 2001 to 2008, remained between 11.97% and 14.59% from 2009 to 2019, and regained the benchmark only in 2020 to 2022. Chad (2004) and Madagascar (2010 and 2015 to 2017) attained the benchmark episodically, whereas South Africa’s sustained attainment began in 2015.

## Discussion

### Principal findings

A quarter century after African heads of state pledged to devote at least 15% of government budgets to health, and across a complete 10-year balanced panel ending in 2023, this study finds the commitment essentially unmet. The average government among the original endorsers devoted about 7% of government expenditure to health, with no meaningful upward trend; 92% of countries never touched the benchmark in any year; only South Africa sustained attainment; and the temporary rise in prioritisation during the pandemic years of 2020 and 2021 had dissipated by 2022. Perhaps most striking, the cross-country distribution has barely moved: apparent catch-up by lower spenders was not robust to correction for regression to the mean, and prioritisation has remained centred at roughly half the target since the Declaration itself. Extending the analysis to 2001 shows that this is not a recent phenomenon; over 23 years, the average within-country trend was essentially zero, and Namibia, the only other country to sustain attainment, lost it after 2008. The Abuja shortfall is therefore not the story of a few laggards dragging down a progressing continent; it is a systemic equilibrium in which health commands a persistently modest claim on public resources across nearly all of Africa.

### Comparison with previous evidence

These findings extend and darken earlier assessments. WHO’s 10-year review found limited progress by 2011, with roughly as many countries moving away from the target as towards it [3], and subsequent regional analyses reached similar conclusions for the period to the mid-2010s [8]. The present study shows that a further decade produced no aggregate improvement in prioritisation. The results also align with global health financing research demonstrating that increases in African government health spending per capita have been driven overwhelmingly by economic growth and expanding budgets rather than by reprioritisation towards health [6, 7]. Our fixed-effects estimate of +0.05 percentage points per year quantifies just how little reprioritisation has occurred within countries once stable national characteristics are accounted for. The transience of the 2020 to 2021 rise is consistent with global analyses warning that pandemic-era health spending gains would be vulnerable to subsequent fiscal consolidation, tightening macroeconomic conditions and rising debt service [25–27].

Two recent analyses corroborate and complement these results. Uzor et al., studying 53 African Union Member States for 2000–2022, similarly found that only South Africa met the benchmark in 2022 and that mean prioritisation had risen by only about half a percentage point since 2001, and attributed differences in prioritisation to political commitment and institutional factors rather than income alone [9]. Our study differs in restricting inference to the governments that made the 2001 commitment, in quantifying the persistence of attainment rather than its status in single years, in estimating within-country trends with formal inference and testing their heterogeneity, and in assessing convergence with methods robust to regression to the mean. Asamani et al. addressed a related question, finding that 27 of 47 countries in the WHO African Region were highly or very highly vulnerable to reductions in official development assistance [10]; our finding that most governments have not raised the domestic priority of health suggests limited capacity to absorb such reductions. Estimates of attainment also depend on the data source: reports drawing on budget allocations have described Rwanda, Botswana and Cabo Verde as consistently meeting or exceeding the target [28], whereas none of these countries sustained a 15% share of executed domestic government expenditure in the harmonised series analysed here, underscoring the value of a consistent, expenditure-based monitoring measure.

### Interpretation and policy implications

Several mechanisms plausibly sustain the low-priority equilibrium documented here. Fiscal space for health in many African countries is constrained by narrow tax bases, informality and, increasingly, debt-service obligations that compete directly with social spending [25, 29]. These pressures have intensified since the end of our study period: external health financing faced abrupt cuts in 2025, African countries faced external debt-service obligations of about US$89 billion in 2025, and countries most dependent on external funds now face compounding vulnerability [10, 28]. Where external assistance finances a substantial share of health services, governments may face weakened incentives to raise the domestic share, a displacement dynamic repeatedly observed in health financing research [6, 7]. Even where budget allocations rise, weak public financial management and chronic under-execution of health budgets mean that actual expenditure, the quantity measured here, falls short of appropriations [30]. The sharp declines observed in Madagascar, Zimbabwe, Malawi, Sudan and Algeria illustrate how quickly health can lose ground when fiscal conditions deteriorate, while the overall upward trajectories of Botswana, Cabo Verde and Namibia show that some middle-income countries can approach the benchmark. Our stratified analyses suggest that neither fragility nor natural-resource intensity alone explains the divergent trends, consistent with evidence that political commitment and institutions, rather than income or resource endowment alone, shape the priority given to health [9].

Four implications follow for policy. First, the Abuja benchmark needs an accountability architecture if it is to function as more than rhetoric. The African Union’s Africa Leadership Meeting agenda, with its emphasis on domestic resource mobilisation, country-led tracking and peer review, provides a vehicle, but our results indicate it must engage the large stagnant middle of 22 countries, not only the worst performers [11]. Second, share-based targets should be complemented by country-specific commitments grounded in need and capacity. Analyses suggest government health spending of about 5% of gross domestic product, or approximately 86 United States dollars per person, as an evidence-based reference for universal health coverage [5]; pairing such absolute anchors with the Abuja share would prevent the paradox in which a shrinking or expanding denominator, rather than health policy, drives measured performance. Third, the finding that actual expenditure shares stagnated even as many governments announced health-sector initiatives points to budget execution and public financial management as decisive reform frontiers, since credibility gaps between approved and executed budgets directly erode the indicator studied here [30]. Earmarking and other revenue instruments can help, but only when designed to add resources rather than substitute for general allocations [31]. Fourth, the stakes of inaction are borne by households: where public financing stagnates, out-of-pocket payment fills the gap, exposing populations to catastrophic health expenditure and undermining universal health coverage commitments reaffirmed by the United Nations in 2019 [12, 32–35].

These continental implications need to be differentiated according to country trajectory (Table 2 and Supplementary Table S1), in the spirit of country-specific financing targets [5]. For South Africa and the four near-target countries (Namibia, Botswana, Cabo Verde and Tunisia), the priority is to protect gains against fiscal shocks, for example through multi-year budget frameworks and protected minimum allocations, and to shift attention towards the efficiency, equity and execution of spending, because the Namibian and Malagasy experiences show that attainment can be lost quickly. For the 16 improving countries, many starting from very low levels, such as the Central African Republic, Equatorial Guinea and the Republic of the Congo, the task is to institutionalise recent increases, so that they survive political transitions and commodity cycles, by embedding them in medium-term expenditure frameworks and tracking executed rather than budgeted spending. For the 22 stagnant countries, including populous countries such as Nigeria and Ethiopia whose shares remained around 4–6%, incremental reprioritisation is unlikely to suffice; explicit country compacts that pair the Abuja share with an absolute per-capita spending anchor [5], stronger domestic revenue mobilisation and well-designed health taxes or earmarks [31] are more promising routes. For the nine declining countries, several of which experienced macroeconomic crises, conflict or debt distress (for example Zimbabwe, Malawi, Sudan and Madagascar), the first objective is to halt erosion by protecting health budget lines during fiscal consolidation, securing health-protective provisions in debt restructuring and lender-supported adjustment programmes, and considering debt-for-health swaps where appropriate [10]. Across all groups, external partners could align transitional support with these trajectories, prioritising countries whose domestic prioritisation is declining and whose dependence on external funds is high [10].

### Strengths and limitations

The study’s strengths include a prespecified population defined by the governments that actually made the 2001 commitment, a strongly balanced 520-observation panel with no missing data, internationally harmonised expenditure estimates produced under the System of Health Accounts 2011 framework, an analytical design that jointly examines level, trend, persistence and convergence with country fixed effects and clustered inference, an extension to the full post-Declaration period, and robustness analyses addressing classification thresholds, regression to the mean and heterogeneous country trends.

Several limitations warrant emphasis. First, the Abuja Declaration refers to the share of the approved national budget allocated to health, whereas our indicator measures the share of government spending actually executed on health from domestic sources. The two can differ: a government could allocate 15% of its budget to health but, because of under-execution, spend less. Our estimates therefore describe realised rather than promised prioritisation. Second, the measure is a ratio, and movements in total government expenditure, for example pandemic stimulus or post-crisis consolidation, can alter the share without any change in health spending itself. Third, Global Health Expenditure Database values incorporate estimation where country reporting is incomplete, and revisions occur across releases; about one in eight country-year values in 2014–2023, and half of all 2023 values, were identical to the preceding year’s value, consistent with carry-forward estimation. Excluding these values did not change the average trend, but classifications based on the most recent year should be interpreted with this in mind, and the longer-term analysis relied on earlier values of similar quality and on incomplete series for Somalia and Zimbabwe. Fourth, analyses were country-weighted and unweighted by population, so results describe the behaviour of governments rather than the financing experienced by the average African resident; population-weighted results would be strongly influenced by a few large countries with low shares, such as Nigeria.

Fifth, the performance-classification thresholds were investigator-defined, although the classification was robust to reasonable alternatives, and the convergence analyses were exploratory; the conventional beta-convergence estimate was partly attributable to regression to the mean. Sixth, as an ecological study without a counterfactual, the analysis describes patterns and associations, including the pandemic-period comparison, and supports no causal claims. Finally, the study was designed to monitor a political commitment rather than to explain it. The heterogeneity documented here, across subregions and between countries with similar fiscal circumstances, provides a basis for analytical studies of the determinants of prioritisation, of the quality and efficiency of public health spending, and of the links between public financing, financial protection and health outcomes, which require designs and data beyond the scope of this study.

## Conclusion

Twenty-five years after the Abuja Declaration, government prioritisation of health across Africa remains locked at less than half the pledged level, with attainment confined to a single sustained achiever and a cross-country distribution that has remained anchored at a low level rather than converging towards the target. Treating the 15% pledge as a living commitment will require enforceable continental accountability, country-specific financing compacts that pair share targets with absolute spending anchors, and public financial management reforms that translate allocations into executed expenditure. Absent such action, the Declaration risks marking its next anniversary as it marks this one: honoured in speeches and unmet in realised spending.

This study adds three elements to the evidence on the Abuja commitment. First, attainment has been not only rare but impermanent: across 23 years, only South Africa has sustained it in recent years, and Namibia, which sustained it earlier, lost it for more than a decade. Second, the average government has made essentially no progress in reprioritising health since 2001, and apparent catch-up by lower spenders was not robust to correction for regression to the mean, pointing to stasis rather than genuine convergence. Third, beneath this stagnant average lie sharply different country trajectories, improving, stagnant and declining, that call for differentiated rather than uniform responses. As external financing contracts and debt-service rises, the gap between the commitment and actual domestic prioritisation has become a direct constraint on sustaining essential services and progressing towards universal health coverage. Regular, consistent, expenditure-based monitoring of the kind presented here, integrated into African Union peer-review mechanisms, would allow governments, parliaments and citizens to hold the commitment to account.

## Supplementary Information

Supplementary Material 1.

## Data Availability

The data analysed in this study are publicly available from the World Bank World Development Indicators under indicator SH.XPD.GHED.GE.ZS, “Domestic general government health expenditure as a percentage of general government expenditure.” The underlying source is the World Health Organisation Global Health Expenditure Database. The country-level analytical dataset derived for this study is available from the corresponding author upon reasonable request.
