# Supplementary material for: Persistent shortfalls in government health expenditure: a decade-long analysis of Abuja 15% target attainment across 52 African countries, 2014–2023
Source: Trop Med Health. 2026 Oct 19;54:222. doi: 10.1186/s41182-026-01102-6 (PMC13679279; doi:10.1186/s41182-026-01102-6)
Supplement: Supplementary file 1 [file 41182_2026_1102_MOESM1_ESM.docx]

**Supplementary material**

**Persistent Shortfalls in Government Health Expenditure: A Decade-Long Analysis of Abuja 15% Target Attainment across 52 African Countries, 2014–2023**

**Contents**

Supplementary Table S1. Country-level government health-expenditure scorecard, 2014–2023

Supplementary Table S2. Annual country-level government health-expenditure shares, 2014–2023

Supplementary Figure S1. Government health-expenditure shares in 2014 versus 2023

Supplementary Table S3. Annual distribution of government health-expenditure shares and benchmark attainment, 2000–2023

Supplementary Table S4. Sensitivity of the 2023 performance classification

Supplementary Table S5. Between-country variation and heterogeneity in country-specific trends, 2014–2023

Supplementary Table S6. Convergence analyses robust to mathematical coupling and regression to the mean

Supplementary Table S7. Country membership of subregions and groups used in subregional and stratified analyses

Supplementary Figure S2. Government health-expenditure shares among original Abuja Declaration endorsers, 2000–2023

**Supplementary Table S1.** *Country-level government health-expenditure scorecard, 2014–2023*

*Original Abuja Declaration endorsers with comparable data (n = 52)*

| **Country** | **2014 share (%)** | **2023 share (%)** | **Absolute change (pp)** | **Annual trend (pp/year)** | **Years meeting 15%, n** | **Longest consecutive attainment, years** | **Persistence category** | **2023 performance category** |
| --- | --- | --- | --- | --- | --- | --- | --- | --- |
| Algeria | 11.42 | 6.17 | -5.25 | -0.610 | 0 | 0 | Never attained | Below target, declining |
| Angola | 3.62 | 5.90 | +2.28 | +0.353 | 0 | 0 | Never attained | Below target, improving |
| Benin | 4.04 | 2.55 | -1.49 | -0.229 | 0 | 0 | Never attained | Below target, declining |
| Botswana | 10.96 | 14.61 | +3.65 | +0.435 | 0 | 0 | Never attained | Near target |
| Burkina Faso | 7.77 | 8.78 | +1.00 | +0.102 | 0 | 0 | Never attained | Below target, stagnant |
| Burundi | 9.79 | 4.73 | -5.06 | -0.615 | 0 | 0 | Never attained | Below target, declining |
| Cabo Verde | 9.98 | 13.79 | +3.81 | +0.679 | 1 | 1 | Occasional attainment | Near target |
| Cameroon | 2.94 | 3.91 | +0.97 | +0.229 | 0 | 0 | Never attained | Below target, improving |
| Central African Republic | 3.95 | 8.48 | +4.53 | +0.511 | 0 | 0 | Never attained | Below target, improving |
| Chad | 5.55 | 7.26 | +1.72 | +0.164 | 0 | 0 | Never attained | Below target, stagnant |
| Comoros | 3.82 | 4.74 | +0.92 | +0.260 | 0 | 0 | Never attained | Below target, improving |
| Cote d'Ivoire | 5.11 | 6.58 | +1.47 | +0.280 | 0 | 0 | Never attained | Below target, improving |
| Democratic Republic of the Congo | 3.99 | 4.00 | +0.01 | +0.039 | 0 | 0 | Never attained | Below target, stagnant |
| Djibouti | 6.07 | 4.89 | -1.18 | -0.088 | 0 | 0 | Never attained | Below target, stagnant |
| Egypt | 4.16 | 6.93 | +2.77 | +0.272 | 0 | 0 | Never attained | Below target, improving |
| Equatorial Guinea | 2.06 | 5.87 | +3.80 | +0.607 | 0 | 0 | Never attained | Below target, improving |
| Eritrea | 2.37 | 2.37 | +0.00 | +0.000 | 0 | 0 | Never attained | Below target, stagnant |
| Eswatini | 8.50 | 10.64 | +2.13 | +0.354 | 0 | 0 | Never attained | Below target, improving |
| Ethiopia | 4.09 | 5.68 | +1.59 | +0.190 | 0 | 0 | Never attained | Below target, stagnant |
| Gabon | 6.38 | 9.59 | +3.21 | +0.294 | 0 | 0 | Never attained | Below target, improving |
| Ghana | 6.72 | 10.05 | +3.33 | +0.159 | 0 | 0 | Never attained | Below target, stagnant |
| Guinea | 3.05 | 5.12 | +2.07 | +0.291 | 0 | 0 | Never attained | Below target, improving |
| Guinea-Bissau | 2.60 | 4.29 | +1.69 | +0.296 | 0 | 0 | Never attained | Below target, improving |
| Kenya | 7.85 | 8.65 | +0.81 | +0.151 | 0 | 0 | Never attained | Below target, stagnant |
| Lesotho | 11.59 | 11.83 | +0.24 | -0.011 | 0 | 0 | Never attained | Below target, stagnant |
| Liberia | 2.46 | 4.79 | +2.33 | +0.178 | 0 | 0 | Never attained | Below target, stagnant |
| Libya | 5.71 | 9.57 | +3.86 | +0.026 | 0 | 0 | Never attained | Below target, stagnant |
| Madagascar | 13.92 | 5.51 | -8.41 | -1.473 | 3 | 3 | Occasional attainment | Below target, declining |
| Malawi | 8.58 | 3.29 | -5.29 | -0.710 | 0 | 0 | Never attained | Below target, declining |
| Mali | 4.45 | 5.43 | +0.98 | +0.092 | 0 | 0 | Never attained | Below target, stagnant |
| Mauritania | 5.24 | 5.94 | +0.70 | +0.096 | 0 | 0 | Never attained | Below target, stagnant |
| Mauritius | 10.53 | 8.24 | -2.29 | -0.169 | 0 | 0 | Never attained | Below target, stagnant |
| Mozambique | 4.32 | 6.76 | +2.45 | +0.250 | 0 | 0 | Never attained | Below target, improving |
| Namibia | 11.97 | 14.99 | +3.03 | +0.430 | 3 | 3 | Occasional attainment | Near target |
| Niger | 5.40 | 6.15 | +0.75 | +0.259 | 0 | 0 | Never attained | Below target, improving |
| Nigeria | 3.34 | 4.31 | +0.97 | -0.013 | 0 | 0 | Never attained | Below target, stagnant |
| Republic of the Congo | 1.95 | 5.42 | +3.47 | +0.483 | 0 | 0 | Never attained | Below target, improving |
| Rwanda | 7.98 | 8.92 | +0.94 | +0.131 | 0 | 0 | Never attained | Below target, stagnant |
| Sao Tome and Principe | 7.78 | 8.77 | +1.00 | +0.394 | 0 | 0 | Never attained | Below target, improving |
| Senegal | 4.68 | 3.57 | -1.10 | -0.119 | 0 | 0 | Never attained | Below target, stagnant |
| Seychelles | 9.54 | 9.58 | +0.05 | -0.006 | 0 | 0 | Never attained | Below target, stagnant |
| Sierra Leone | 7.58 | 4.47 | -3.11 | -0.162 | 0 | 0 | Never attained | Below target, stagnant |
| Somalia | 4.11 | 7.90 | +3.79 | +0.203 | 0 | 0 | Never attained | Below target, improving |
| South Africa | 14.92 | 16.89 | +1.97 | +0.255 | 9 | 9 | Sustained attainment | Met Abuja target |
| Sudan | 10.62 | 7.15 | -3.47 | -0.411 | 0 | 0 | Never attained | Below target, declining |
| Tanzania | 6.94 | 5.14 | -1.80 | -0.408 | 0 | 0 | Never attained | Below target, declining |
| The Gambia | 5.75 | 5.46 | -0.30 | +0.094 | 0 | 0 | Never attained | Below target, stagnant |
| Togo | 4.56 | 2.56 | -2.00 | -0.271 | 0 | 0 | Never attained | Below target, declining |
| Tunisia | 13.54 | 12.10 | -1.44 | -0.115 | 0 | 0 | Never attained | Near target |
| Uganda | 7.01 | 4.86 | -2.15 | -0.098 | 0 | 0 | Never attained | Below target, stagnant |
| Zambia | 7.78 | 8.89 | +1.10 | +0.189 | 0 | 0 | Never attained | Below target, stagnant |
| Zimbabwe | 10.93 | 4.74 | -6.19 | -0.491 | 0 | 0 | Never attained | Below target, declining |

*Notes.* Expenditure share is domestic general government health expenditure as a percentage of general government expenditure. Absolute change is the 2023 value minus the 2014 value, in percentage points (pp). Annual trend is the country-specific linear change in expenditure share across 2014–2023, in percentage points per year. Persistence categories: never attained (0 years at ≥15%), occasional attainment (1–3 years) and sustained attainment (≥8 years); no country fell in the repeated-attainment category (4–7 years). The 2023 performance categories: met Abuja target (≥15%); near target (12% to <15%); below target, improving (<12% and trend ≥+0.20 pp/year); below target, broadly stagnant (<12% and trend between −0.20 and +0.20 pp/year); and below target, declining (<12% and trend ≤−0.20 pp/year). The ±0.20 pp/year thresholds were investigator-defined. Source: World Bank World Development Indicators, indicator SH.XPD.GHED.GE.ZS; underlying source, WHO Global Health Expenditure Database; data retrieved 25 July 2026. Morocco and South Sudan were excluded because they were not original 2001 endorsers; the Sahrawi Arab Democratic Republic had no separate standardised series.

**Supplementary Table S2.** *Annual country-level government health-expenditure shares, 2014–2023*

*Original Abuja Declaration endorsers with comparable data (n = 52)*

| **Country** | **2014** | **2015** | **2016** | **2017** | **2018** | **2019** | **2020** | **2021** | **2022** | **2023** |
| --- | --- | --- | --- | --- | --- | --- | --- | --- | --- | --- |
| Algeria | 11.42 | 10.60 | 10.61 | 10.61 | 10.61 | 8.83 | 8.83 | 8.83 | 5.25 | 6.17 |
| Angola | 3.62 | 4.56 | 5.43 | 5.36 | 5.36 | 5.51 | 7.31 | 8.78 | 6.70 | 5.90 |
| Benin | 4.04 | 3.25 | 4.63 | 3.97 | 4.02 | 3.12 | 2.68 | 1.68 | 2.55 | 2.55 |
| Botswana | 10.96 | 10.42 | 11.21 | 14.32 | 11.54 | 12.01 | 11.66 | 14.61 | 14.61 | 14.61 |
| Burkina Faso | 7.77 | 7.15 | 11.03 | 10.24 | 8.79 | 9.58 | 11.65 | 9.84 | 8.40 | 8.78 |
| Burundi | 9.79 | 10.44 | 9.90 | 8.36 | 7.91 | 7.91 | 7.33 | 7.33 | 4.73 | 4.73 |
| Cabo Verde | 9.98 | 10.09 | 10.39 | 9.12 | 11.27 | 11.78 | 13.45 | 14.89 | 16.06 | 13.79 |
| Cameroon | 2.94 | 1.81 | 1.81 | 0.73 | 1.96 | 2.18 | 3.66 | 2.86 | 3.91 | 3.91 |
| Central African Republic | 3.95 | 3.26 | 4.95 | 5.26 | 3.91 | 4.84 | 4.86 | 6.42 | 8.48 | 8.48 |
| Chad | 5.55 | 5.25 | 5.73 | 4.68 | 4.86 | 4.86 | 3.95 | 5.64 | 7.26 | 7.26 |
| Comoros | 3.82 | 2.45 | 3.04 | 3.59 | 3.99 | 4.13 | 6.89 | 4.74 | 4.74 | 4.74 |
| Cote d'Ivoire | 5.11 | 4.87 | 4.85 | 5.15 | 5.12 | 5.60 | 6.54 | 7.77 | 6.82 | 6.58 |
| Democratic Republic of the Congo | 3.99 | 4.10 | 3.81 | 3.84 | 4.17 | 4.13 | 5.09 | 4.51 | 3.98 | 4.00 |
| Djibouti | 6.07 | 4.07 | 5.14 | 4.80 | 5.17 | 5.16 | 4.16 | 3.32 | 5.10 | 4.89 |
| Egypt | 4.16 | 5.06 | 5.06 | 5.42 | 4.72 | 4.82 | 5.16 | 6.80 | 6.76 | 6.93 |
| Equatorial Guinea | 2.06 | 1.79 | 3.11 | 3.94 | 4.28 | 4.79 | 6.40 | 9.00 | 5.86 | 5.87 |
| Eritrea | 2.37 | 2.37 | 2.37 | 2.37 | 2.37 | 2.37 | 2.37 | 2.37 | 2.37 | 2.37 |
| Eswatini | 8.50 | 8.59 | 8.22 | 9.97 | 9.70 | 12.93 | 10.39 | 12.30 | 10.64 | 10.64 |
| Ethiopia | 4.09 | 5.57 | 5.02 | 4.79 | 4.76 | 4.76 | 6.79 | 7.10 | 5.68 | 5.68 |
| Gabon | 6.38 | 7.03 | 9.20 | 9.72 | 9.42 | 9.59 | 9.59 | 9.59 | 9.59 | 9.59 |
| Ghana | 6.72 | 8.56 | 6.54 | 9.27 | 6.56 | 8.77 | 8.02 | 7.71 | 7.41 | 10.05 |
| Guinea | 3.05 | 1.98 | 4.13 | 3.48 | 3.88 | 6.19 | 4.55 | 4.51 | 5.12 | 5.12 |
| Guinea-Bissau | 2.60 | 2.50 | 2.36 | 2.75 | 2.59 | 2.44 | 4.80 | 4.55 | 4.88 | 4.29 |
| Kenya | 7.85 | 8.07 | 8.03 | 6.79 | 7.22 | 8.23 | 8.91 | 9.28 | 8.65 | 8.65 |
| Lesotho | 11.59 | 10.86 | 10.34 | 11.97 | 10.78 | 12.61 | 8.95 | 8.15 | 12.88 | 11.83 |
| Liberia | 2.46 | 3.24 | 3.90 | 3.96 | 5.17 | 4.20 | 3.75 | 3.73 | 4.79 | 4.79 |
| Libya | 5.71 | 7.51 | 9.91 | 8.86 | 9.27 | 8.36 | 9.74 | 6.91 | 5.06 | 9.57 |
| Madagascar | 13.92 | 15.27 | 17.51 | 15.03 | 10.43 | 4.44 | 4.22 | 5.38 | 5.51 | 5.51 |
| Malawi | 8.58 | 9.73 | 9.68 | 8.19 | 8.71 | 7.94 | 6.87 | 5.76 | 3.29 | 3.29 |
| Mali | 4.45 | 4.39 | 5.43 | 5.14 | 5.67 | 4.84 | 5.37 | 5.04 | 5.60 | 5.43 |
| Mauritania | 5.24 | 5.53 | 5.76 | 6.07 | 4.80 | 6.18 | 5.42 | 7.20 | 5.94 | 5.94 |
| Mauritius | 10.53 | 9.46 | 10.20 | 10.20 | 10.21 | 9.00 | 8.99 | 10.90 | 8.61 | 8.24 |
| Mozambique | 4.32 | 5.55 | 6.00 | 6.07 | 5.73 | 6.52 | 7.06 | 7.11 | 6.96 | 6.76 |
| Namibia | 11.97 | 12.35 | 13.80 | 14.02 | 14.21 | 14.08 | 15.71 | 16.60 | 15.88 | 14.99 |
| Niger | 5.40 | 4.59 | 5.69 | 9.66 | 8.35 | 9.38 | 10.34 | 8.75 | 7.11 | 6.15 |
| Nigeria | 3.34 | 5.33 | 4.87 | 4.44 | 3.88 | 3.76 | 4.18 | 4.31 | 4.31 | 4.31 |
| Republic of the Congo | 1.95 | 2.71 | 2.89 | 3.41 | 3.49 | 5.11 | 8.22 | 8.22 | 3.53 | 5.42 |
| Rwanda | 7.98 | 7.91 | 6.23 | 6.23 | 6.23 | 6.28 | 6.39 | 8.35 | 8.20 | 8.92 |
| Sao Tome and Principe | 7.78 | 4.94 | 8.00 | 10.76 | 9.39 | 9.78 | 12.80 | 13.14 | 8.34 | 8.77 |
| Senegal | 4.68 | 4.70 | 4.46 | 4.50 | 4.96 | 3.50 | 5.85 | 3.88 | 3.34 | 3.57 |
| Seychelles | 9.54 | 9.90 | 9.45 | 9.55 | 9.58 | 9.58 | 9.58 | 9.58 | 9.58 | 9.58 |
| Sierra Leone | 7.58 | 6.21 | 4.45 | 3.63 | 3.60 | 5.84 | 5.30 | 5.67 | 4.47 | 4.47 |
| Somalia | 4.11 | 5.25 | 5.78 | 4.73 | 4.08 | 4.14 | 4.21 | 7.12 | 4.42 | 7.90 |
| South Africa | 14.92 | 15.24 | 15.29 | 15.29 | 15.29 | 15.29 | 16.89 | 16.89 | 16.89 | 16.89 |
| Sudan | 10.62 | 6.66 | 12.93 | 8.32 | 6.17 | 5.56 | 9.55 | 7.86 | 4.62 | 7.15 |
| Tanzania | 6.94 | 7.33 | 9.52 | 9.52 | 9.41 | 9.44 | 4.94 | 5.14 | 5.14 | 5.14 |
| The Gambia | 5.75 | 5.10 | 6.66 | 4.49 | 5.51 | 5.38 | 7.70 | 5.98 | 6.83 | 5.46 |
| Togo | 4.56 | 4.04 | 4.26 | 6.45 | 5.49 | 5.38 | 3.85 | 2.56 | 2.56 | 2.56 |
| Tunisia | 13.54 | 12.18 | 12.52 | 11.56 | 10.78 | 12.08 | 12.49 | 12.39 | 10.84 | 12.10 |
| Uganda | 7.01 | 5.08 | 5.18 | 3.92 | 4.17 | 3.14 | 5.55 | 5.35 | 4.86 | 4.86 |
| Zambia | 7.78 | 7.56 | 7.16 | 10.05 | 7.53 | 7.57 | 10.56 | 9.25 | 8.89 | 8.89 |
| Zimbabwe | 10.93 | 6.21 | 5.49 | 6.98 | 7.98 | 3.27 | 4.74 | 4.74 | 4.74 | 4.74 |

*Notes.* Values are domestic general government health expenditure as a percentage of general government expenditure. The table includes 52 original Abuja Declaration endorsers with complete comparable data for 2014–2023. The Abuja benchmark is 15%. Values are presented to two decimal places; analyses used unrounded values. Source: World Bank World Development Indicators, indicator SH.XPD.GHED.GE.ZS; underlying source, WHO Global Health Expenditure Database; data retrieved 25 July 2026. Morocco and South Sudan were excluded because they were not original 2001 endorsers; the Sahrawi Arab Democratic Republic had no separate standardised series.

**Supplementary Figure S1.** *Government health-expenditure shares in 2014 versus 2023 among original Abuja Declaration endorsers*

Each point represents one of the 52 original Abuja Declaration endorsers with comparable data, plotting the 2014 domestic general government health expenditure share (horizontal axis) against the 2023 share (vertical axis). The 45-degree line indicates no endpoint change: countries above the line increased their expenditure share between 2014 and 2023, while countries below the line declined. The horizontal and vertical dashed lines at 15% indicate Abuja benchmark attainment at each endpoint. Source: World Bank World Development Indicators, indicator SH.XPD.GHED.GE.ZS; underlying source, WHO Global Health Expenditure Database.

**Supplementary Table S3.** *Annual distribution of government health-expenditure shares and benchmark attainment, 2000–2023*

*Original Abuja Declaration endorsers with available data*

| **Year** | **Countries, n** | **Mean (%)** | **Median (%)** | **IQR (%)** | **SD (pp)** | **≥15%, n** | **Countries meeting 15%** |
| --- | --- | --- | --- | --- | --- | --- | --- |
| 2000 (pre-Declaration) | 50 | 7.15 | 6.67 | 4.43–8.37 | 4.91 | 2 | Namibia; Sao Tome and Principe |
| 2001 | 50 | 6.88 | 6.65 | 4.46–8.80 | 3.22 | 1 | Namibia |
| 2002 | 50 | 6.90 | 6.44 | 4.44–8.54 | 3.23 | 1 | Namibia |
| 2003 | 50 | 7.33 | 6.90 | 4.26–9.29 | 3.51 | 1 | Namibia |
| 2004 | 50 | 7.26 | 6.84 | 5.26–8.97 | 3.44 | 2 | Chad; Namibia |
| 2005 | 50 | 7.15 | 6.40 | 5.24–8.54 | 3.50 | 1 | Namibia |
| 2006 | 50 | 6.89 | 6.64 | 5.05–8.18 | 3.50 | 1 | Namibia |
| 2007 | 50 | 6.77 | 6.29 | 4.33–8.18 | 3.48 | 1 | Namibia |
| 2008 | 50 | 6.37 | 5.64 | 4.06–8.08 | 3.23 | 1 | Namibia |
| 2009 | 50 | 6.38 | 5.80 | 4.09–7.63 | 3.25 | 0 | – |
| 2010 | 51 | 6.65 | 6.03 | 4.10–8.56 | 3.56 | 1 | Madagascar |
| 2011 | 51 | 6.69 | 6.59 | 4.31–8.30 | 3.43 | 0 | – |
| 2012 | 51 | 6.72 | 6.11 | 4.22–8.86 | 3.27 | 0 | – |
| 2013 | 52 | 6.51 | 5.98 | 4.51–8.19 | 3.05 | 0 | – |
| 2014 | 52 | 6.73 | 5.91 | 4.07–9.06 | 3.36 | 0 | – |
| 2015 | 52 | 6.51 | 5.54 | 4.25–8.57 | 3.31 | 2 | Madagascar; South Africa |
| 2016 | 52 | 7.11 | 5.77 | 4.74–9.79 | 3.52 | 2 | Madagascar; South Africa |
| 2017 | 52 | 7.15 | 6.15 | 4.47–9.69 | 3.50 | 2 | Madagascar; South Africa |
| 2018 | 52 | 6.83 | 5.70 | 4.22–9.40 | 3.08 | 1 | South Africa |
| 2019 | 52 | 6.87 | 5.72 | 4.60–9.19 | 3.24 | 1 | South Africa |
| 2020 | 52 | 7.39 | 6.83 | 4.83–9.56 | 3.29 | 2 | Namibia; South Africa |
| 2021 | 52 | 7.51 | 7.12 | 4.89–9.13 | 3.53 | 2 | Namibia; South Africa |
| 2022 | 52 | 6.90 | 5.77 | 4.73–8.44 | 3.45 | 3 | Cabo Verde; Namibia; South Africa |
| 2023 | 52 | 7.09 | 6.05 | 4.77–8.83 | 3.30 | 1 | South Africa |

Notes. Values are unweighted country-level summaries of domestic general government health expenditure as a percentage of general government expenditure (World Bank indicator SH.XPD.GHED.GE.ZS; underlying source WHO Global Health Expenditure Database). Data were available for 50 countries in 2000–2009 (no values for Somalia or Zimbabwe), 51 in 2010–2012 (Zimbabwe added) and all 52 from 2013. The 2000 value for Sao Tome and Principe (32.57%) is an outlier in the source series; 2000 is shown only as a pre-Declaration reference. Longer-term models used the 50 countries with complete data for 2001–2023. Country fixed-effects linear trend, 2001–2023: +0.012 percentage points per year (95% CI −0.032 to +0.057; p = 0.572). Piecewise model: slope before 2014 −0.037 (95% CI −0.103 to +0.029; p = 0.268); slope from 2014 +0.096 (95% CI +0.002 to +0.191; p = 0.046); change in slope +0.133 (95% CI 0.000 to +0.266; p = 0.049). Over 2001–2023, 47 of 52 countries never reached 15% in any year with available data; Namibia reached it in 11 years (longest run 8 years, 2001–2008), South Africa in 9 (2015–2023), Madagascar in 4 (longest run 3 years), and Chad and Cabo Verde in 1 year each. IQR, interquartile range (25th–75th percentile); SD, standard deviation; pp, percentage points.

**Supplementary Table S4.** *Sensitivity of the 2023 performance classification to alternative thresholds, trend estimators and reference year*

| **Specification** | **Met** | **Near** | **Improving** | **Stagnant** | **Declining** | **Agreement with primary (%)** | **Cohen’s κ** |
| --- | --- | --- | --- | --- | --- | --- | --- |
| Primary specification: near target 12%; trend ±0.20 pp/year; OLS slope | 1 | 4 | 16 | 22 | 9 | 100.0 | 1.00 |
| Near-target threshold 10% | 1 | 7 | 15 | 20 | 9 | 94.2 | 0.92 |
| Near-target threshold 13% | 1 | 3 | 16 | 23 | 9 | 98.1 | 0.97 |
| Trend threshold ±0.10 pp/year | 1 | 4 | 24 | 11 | 12 | 78.8 | 0.71 |
| Trend threshold ±0.15 pp/year | 1 | 4 | 22 | 14 | 11 | 84.6 | 0.78 |
| Trend threshold ±0.25 pp/year | 1 | 4 | 13 | 26 | 8 | 92.3 | 0.89 |
| Trend threshold ±0.30 pp/year | 1 | 4 | 6 | 34 | 7 | 76.9 | 0.65 |
| Theil–Sen slope (±0.20 pp/year) | 1 | 4 | 14 | 23 | 10 | 94.2 | 0.92 |
| Endpoint-average trend: (mean 2021–2023 − mean 2014–2016)/7 (±0.20) | 1 | 4 | 16 | 22 | 9 | 96.2 | 0.94 |
| Inferential rule: improving/declining if slope 95% CI excludes zero | 1 | 4 | 14 | 27 | 6 | 86.5 | 0.80 |
| 2022 as reference year (2022 share; 2014–2022 OLS trend; ±0.20) | 3 | 2 | 17 | 21 | 9 | 86.5 | 0.81 |

Notes. Cells show numbers of countries (n = 52). Categories: met (≥15%); near target (primary 12% to <15%); below target and improving, broadly stagnant or declining according to the trend rule. Rationale for primary cut points: 12% corresponds to 80% of the benchmark; ±0.20 pp/year corresponds to about 2 pp over the decade, roughly one quarter of the average 2023 gap to 15% (7.9 pp) and about half the between-country SD of country slopes (0.38 pp/year). Under every specification at least 23 countries were below target and not improving. Countries reclassified at ±0.10: Burkina Faso, Chad, Ethiopia, Ghana, Kenya, Liberia, Rwanda and Zambia (stagnant to improving) and Mauritius, Senegal and Sierra Leone (stagnant to declining). At ±0.30: Cameroon, Comoros, Cote d'Ivoire, Egypt, Gabon, Guinea, Guinea-Bissau, Mozambique, Niger and Somalia (improving to stagnant) and Benin and Togo (declining to stagnant). With 2022 as reference year, Cabo Verde and Namibia moved from near target to met, Lesotho from stagnant to near target, Tunisia from near target to stagnant, Ethiopia and Zambia from stagnant to improving, and Somalia from improving to stagnant. Comparison with quintiles: all 9 declining countries were in the lowest quintile of country-specific trends (−1.47 to −0.16 pp/year) and all 16 improving countries in the top two quintiles (≥0.19 pp/year). Quintiles of the 2023 share are not anchored to the benchmark: the top quintile spanned 9.57% to 16.89%, grouping, for example, Libya with South Africa. OLS, ordinary least squares; pp, percentage points; SD, standard deviation.

**Supplementary Table S5.** *Between-country variation and heterogeneity in country-specific trends, 2014–2023*

| **Measure** | **Estimate** | **p value** |
| --- | --- | --- |
| **Variation in levels** | | |
| Range of 2023 shares | 2.37% (Eritrea) to 16.89% (South Africa) | – |
| Between-country SD of country means, pp | 3.01 | – |
| Within-country SD (deviations from country means), pp | 1.51 | – |
| Share of total variance between countries (intraclass correlation) | 0.78 | – |
| **Heterogeneity of country-specific trends** | | |
| Country-specific OLS slopes, median (IQR), pp/year | +0.141 (−0.102 to +0.274) | – |
| Range of country-specific slopes, pp/year | −1.473 (Madagascar) to +0.679 (Cabo Verde) | – |
| Countries with slope 95% CI above / below zero, n | 18 / 6 | – |
| F test of equal country-specific slopes | F(51, 416) = 8.33 | <0.001 |
| Mixed model: mean slope, pp/year (95% CI) | +0.053 (−0.042 to +0.147) | – |
| Mixed model: SD of random slopes, pp/year | 0.323 | <0.001 (LR test) |
| **Trends by subgroup, pp/year (95% CI)** | | |
| Eastern Africa (n = 17) | −0.158 (−0.394 to +0.079) | 0.176 |
| Middle Africa (n = 9) | +0.341 (+0.202 to +0.481) | <0.001 |
| Northern Africa (n = 5) | −0.168 (−0.606 to +0.271) | 0.348 |
| Southern Africa (n = 5) | +0.292 (+0.061 to +0.524) | 0.025 |
| Western Africa (n = 16) | +0.108 (−0.019 to +0.236) | 0.090 |
| Joint test of subregion × year interactions | F(4, 51) = 6.79 | <0.001 |
| Fragile and conflict-affected (n = 20) | +0.098 (−0.034 to +0.231) | – |
| Not fragile or conflict-affected (n = 32) | +0.024 (−0.123 to +0.172) | 0.742 |
| Difference (fragile − other) | +0.074 (−0.124 to +0.273) | 0.457 |
| Resource-intensive (n = 26) | +0.135 (+0.019 to +0.251) | – |
| Non-resource-intensive (n = 26) | −0.029 (−0.198 to +0.139) | 0.728 |
| Difference (resource-intensive − other) | +0.164 (−0.040 to +0.368) | 0.113 |
| Oil exporters (n = 9) | +0.170 (−0.056 to +0.396) | – |
| Difference (oil exporters − other) | +0.142 (−0.112 to +0.396) | 0.268 |
| **Sensitivity to carried-forward values** | | |
| Linear trend excluding values identical to the preceding year (n = 455 country-years) | +0.070 (−0.039 to +0.179) | 0.201 |

Notes. Subgroup trends were estimated with country fixed effects, group × year interactions and standard errors clustered by country; p values for single groups test a zero slope and those for differences test equality of slopes. The mixed model included country random intercepts and random slopes for calendar year (maximum likelihood); the likelihood-ratio (LR) test compared it with a random-intercept model using a 50:50 mixture of χ²(1) and χ²(2). The mixed-model mean slope equals the fixed-effects estimate in the main analysis (+0.053), which therefore represents the average of heterogeneous country trends. Country-specific slopes and confidence intervals from separate OLS regressions (10 observations each) are descriptive. Country membership of each subregion and group is listed in Supplementary Table S7. In 2014–2023, 65 of 520 country-year values (12.5%) and 26 of 52 values for 2023 were identical to the preceding year’s value. IQR, interquartile range; OLS, ordinary least squares; pp, percentage points; SD, standard deviation.

**Supplementary Table S6.** *Convergence analyses robust to mathematical coupling and regression to the mean*

| **Analysis** | **Estimate** | **95% CI** | **p value** |
| --- | --- | --- | --- |
| **Beta convergence** | | | |
| Conventional: 2014–2023 change on 2014 share (primary) | −0.382 | −0.667 to −0.097 | 0.010 |
| Independent baseline: 2014–2023 change on mean 2011–2013 share | −0.247 | −0.570 to +0.076 | 0.131 |
| Independent baseline, averaged endpoints: (mean 2021–2023 − mean 2014–2016) on mean 2011–2013 share | −0.202 | −0.527 to +0.122 | 0.216 |
| Instrumental variable: 2014 share instrumented by mean 2011–2013 share (first-stage F = 177) | −0.253 | −0.554 to +0.047 | 0.098 |
| Oldham’s method: correlation of 2014–2023 change with mean of 2014 and 2023 shares (r) | −0.020 | −0.291 to +0.254 | 0.887 |
| **Sigma convergence** | | | |
| Between-country SD, 2014 and 2023, pp | 3.36 and 3.30 | – | – |
| Bootstrap change in SD, 2014 to 2023, pp | −0.052 | −0.734 to +0.523 | 0.888 |
| Bootstrap change in IQR, 2014 to 2023, pp | −0.927 | −2.938 to +0.699 | 0.391 |
| Annual change in SD, 2014–2023 (10 years; primary) | +0.001 | −0.024 to +0.025 | 0.933 |
| Annual change in IQR, 2014–2023 (10 years; primary) | −0.114 | −0.214 to −0.015 | 0.030 |
| Annual change in SD, 2001–2023 (23 years; 50 countries) | +0.001 | −0.009 to +0.011 | 0.828 |
| Annual change in IQR, 2001–2023 (23 years; 50 countries) | +0.030 | −0.019 to +0.079 | 0.214 |

Notes. In the conventional beta-convergence model the 2014 value enters both sides of the regression, so measurement error and transitory fluctuations in 2014 induce a negative association even without true convergence (regression to the mean; Galton’s fallacy). The independent-baseline and instrumental-variable specifications use the 2011–2013 mean, measured separately from the change score; Oldham’s method tests whether change is related to the average level and is equivalent to testing equality of the 2014 and 2023 variances. Beta regressions used heteroskedasticity-robust standard errors (n = 52). Bootstrap estimates used 5,000 country-resampling replicates (percentile intervals; two-sided bootstrap p values). Sigma regressions for 2001–2023 used Newey–West standard errors (2 lags). IQR, interquartile range; pp, percentage points; SD, standard deviation.

**Supplementary Table S7.** *Country membership of subregions and groups used in subregional and stratified analyses*

| **Group** | **Countries** |
| --- | --- |
| Eastern Africa, n = 17 | Burundi, Comoros, Djibouti, Eritrea, Ethiopia, Kenya, Madagascar, Malawi, Mauritius, Mozambique, Rwanda, Seychelles, Somalia, Tanzania, Uganda, Zambia, Zimbabwe. |
| Middle Africa, n = 9 | Angola, Cameroon, Central African Republic, Chad, Democratic Republic of the Congo, Equatorial Guinea, Gabon, Republic of the Congo, Sao Tome and Principe. |
| Northern Africa, n = 5 | Algeria, Egypt, Libya, Sudan, Tunisia. |
| Southern Africa, n = 5 | Botswana, Eswatini, Lesotho, Namibia, South Africa. |
| Western Africa, n = 16 | Benin, Burkina Faso, Cabo Verde, Cote d'Ivoire, Ghana, Guinea, Guinea-Bissau, Liberia, Mali, Mauritania, Niger, Nigeria, Senegal, Sierra Leone, The Gambia, Togo. |
| Fragile and conflict-affected situations (World Bank FY24 list), n = 20 | Conflict: Burkina Faso, Cameroon, Central African Republic, Democratic Republic of the Congo, Ethiopia, Mali, Mozambique, Niger, Nigeria, Somalia, Sudan. Institutional and social fragility: Burundi, Chad, Comoros, Eritrea, Guinea-Bissau, Libya, Republic of the Congo, Sao Tome and Principe, Zimbabwe. |
| Not fragile or conflict-affected, n = 32 | Algeria, Angola, Benin, Botswana, Cabo Verde, Cote d'Ivoire, Djibouti, Egypt, Equatorial Guinea, Eswatini, Gabon, Ghana, Guinea, Kenya, Lesotho, Liberia, Madagascar, Malawi, Mauritania, Mauritius, Namibia, Rwanda, Senegal, Seychelles, Sierra Leone, South Africa, Tanzania, The Gambia, Togo, Tunisia, Uganda, Zambia. |
| Resource-intensive (IMF classification), n = 26 | Oil exporters (n = 9): Algeria, Angola, Cameroon, Chad, Equatorial Guinea, Gabon, Libya, Nigeria, Republic of the Congo. Other resource-intensive (n = 17): Botswana, Burkina Faso, Central African Republic, Democratic Republic of the Congo, Eritrea, Ghana, Guinea, Liberia, Mali, Mauritania, Namibia, Niger, Sierra Leone, South Africa, Tanzania, Zambia, Zimbabwe. |
| Non-resource-intensive, n = 26 | Benin, Burundi, Cabo Verde, Comoros, Cote d'Ivoire, Djibouti, Egypt, Eswatini, Ethiopia, Guinea-Bissau, Kenya, Lesotho, Madagascar, Malawi, Mauritius, Mozambique, Rwanda, Sao Tome and Principe, Senegal, Seychelles, Somalia, Sudan, The Gambia, Togo, Tunisia, Uganda. |

Notes. Subregions follow the United Nations Statistics Division M49 classification. Fragile and conflict-affected status follows the World Bank list for fiscal year 2024, which covers the end of the study period. Resource intensity follows the International Monetary Fund Regional Economic Outlook for Sub-Saharan Africa (October 2023): oil exporters are countries where net oil exports make up 30% or more of total exports, and other resource-intensive countries are those where non-renewable natural resources represent 25% or more of total exports. For countries outside that grouping, Algeria and Libya were classified as oil exporters and Mauritania, whose exports are dominated by iron ore and gold, as resource-intensive; Djibouti, Egypt, Somalia, Sudan and Tunisia were classified as non-resource-intensive.

**Supplementary Figure S2.** *Government health-expenditure shares among original Abuja Declaration endorsers, 2000–2023*

Panel A shows the annual cross-country median (squares), interquartile range (shaded band) and unweighted mean (circles, dashed) of domestic general government health expenditure as a percentage of general government expenditure among original Abuja Declaration endorsers with available data (50 countries in 2000–2009, 51 in 2010–2012 and 52 from 2013). The vertical line marks the April 2001 Declaration, the dotted horizontal line the 15% benchmark, and the grey shading the primary analysis window (2014–2023). Panel B shows trajectories for selected countries: those that met or approached the benchmark (South Africa, Namibia, Botswana, Cabo Verde, Tunisia), those illustrating reversal (Madagascar, Chad), and the most populous endorser (Nigeria). Red points mark years at or above 15%; the grey line is the all-country median. Source: World Bank World Development Indicators, indicator SH.XPD.GHED.GE.ZS; underlying source, WHO Global Health Expenditure Database.
